# Which feature influences on-eye power change of soft toric contact lenses: Design or corneal shape?

**DOI:** 10.1371/journal.pone.0242243

**Published:** 2020-11-25

**Authors:** Tamsin Doll, Joshua Moore, Ahmad H. Shihab, Bernardo T. Lopes, Ashkan Eliasy, Osama Maklad, Richard Wu, Lynn White, Steve Jones, Ahmed Elsheikh, Ahmed Abass

**Affiliations:** 1 School of Engineering, University of Liverpool, Liverpool, United Kingdom; 2 Department of Mathematical Sciences, University of Liverpool, Liverpool, United Kingdom; 3 School of Engineering, University of Hertfordshire, Hatfield, United Kingdom; 4 Department of Ophthalmology, Federal University of Sao Paulo, Sao Paulo, Brazil; 5 Department of Optometry, Central Taiwan University of Science and Technology, Taichung, Taiwan; 6 College of Optometry, Pacific University, Forest Grove, Oregon, United States of America; 7 Ultravision CLPL, Leighton Buzzard, United Kingdom; 8 School of Biological Science and Biomedical Engineering, Beihang University, Beijing, China; 9 National Institute for Health Research (NIHR) Biomedical Research Centre at Moorfields Eye Hospital NHS Foundation Trust and UCL Institute of Ophthalmology, London, United Kingdom; Cardiff University, UNITED KINGDOM

## Abstract

**Purpose:**

This study investigates how both the peripheral zone design and corneal shape affect the behaviour of soft contact lenses on-eye.

**Methods:**

In this study, soft contact lenses of varying nominal cylindrical powers and peripheral zone designs—a single-prism gravity-based stabilised lens (G1P), two-prism blink-based stabilised lens (B2P) and four-prism blink-based stabilised lens (B4P)—were generated as finite element models. The on-eye simulation results were analysed to identify the impact of each peripheral zone design (Each with different volume ratios) on the effective power change (EPC) when worn by a subject. Topographies of three eyes of varying average simulated anterior corneal curvature (flat, average & steep) were used in this study.

**Results:**

The volume of the lens’s peripheral zone as a ratio of the total lens volume (V_p_) recorded very weak correlations with the effective power change (EPC) among the three investigated designs when they were fitted to the flat eye (R = -0.19, -0.15 & -0.22 respectively), moderate correlations with the average eye (R = 0.42, 0.43 & 0.43 respectively) and strong correlations with the steep eye (R = 0.91, 0.9 & 0.9 respectively). No significant differences were noticed among the three investigated designs and none of the cylindrical lenses designed with axis 90° recorded EPC values outside the acceptance criteria range (ACR) of ±0.25 D. No significant differences in EPC were recorded among the three designs G1P, B2P and B4P (p>0.6) when they were designed with three axes at 90°, 45° and 0°. Moving the toric lens axis away from 90° dragged the EPC to the negative side and most of the investigated lenses with axes at 45° and 0° recorded EPCs outside the ±0.25D range.

**Conclusions:**

In all cases, the shape of the cornea had a more dominant effect on EPC when compared to the peripheral zone design. Corneal shape influences the soft toric contact lens’s on-eye power change more than the lens design.

## Introduction

Contact lenses are medical devices worn by over 150 million people worldwide [1]. Soft-structured contact lenses consist of two main zones; the optic zone designed to achieve the required refractive power that have few parameters for adjustment, and the peripheral zone which is designed to keep the lens on the eye using several geometric parameters [2]. In soft contact lens design, the peripheral zone is a specific region connecting the optic zone to the edge profile. This zone is substantial in contact lens fitting; it has been shown that the peripheral corneal shape has a more significant role in successful contact lens wear than the central radius of curvature of the cornea [3, 4]. Also when a soft contact lens is fitted to a cornea, it is the peripheral zone that flexes and deforms most [5] which may then influence the optic zone, to which it is connected. Any deformation of the optic zone will, in turn, affect the optical power profile. This change in lens power is termed effective power change (EPC).

Mostly, soft lenses fitting approaches lack scientific basis compared to Rigid Gas Permeable (GP) lenses [6]. In the published literature, designers did not go into the scientific details of why certain soft lenses design patterns work better than others. The actual physical characteristics of the lens were either overlooked when calculating refractive optical power [7, 8] or simplified to theoretical statements of flexure hypotheses or empirical models [9–16].

In spherical soft contact lens designs, the peripheral zone is normally rotationally symmetrical and has a uniform thickness in all polar directions, thus changes are expected to be transmitted almost symmetrically. However, in toric lenses, the design requirement to avoid rotation [17] has led to the design of various stabilisation methods [18] which result in non-symmetrical areas of thickness in the peripheral zone. As the regional increased thickness influences the mechanical behaviour of the peripheral zone. It follows that the central optic zone also flexes in different ways depending on the stabilisation design, which affects the optical power profile. The question arises as to whether this change would affect the lens’s refractive power significantly.

To determine the EPC in soft contact lenses when worn on-eye, a few techniques have evolved in the literature [5]. Strachan et al [6] proposed a technique to demonstrate the effect of lens geometry on the power of the lens on-eye. To achieve this, they used the ratio between the lens base curve and the radius of the cornea. For a more comprehensive review of the optical properties used in contact lens design, the reader is referred to Whittle et al [19].

Current market trends show an overall move towards disposable lenses [20]. Although some monthly lenses have a relatively wide parameter range, daily disposable lenses are constrained by the method of manufacture (injection moulding), and often offer no more than two base curves and one diameter—in fact, most brands only offer one base curve. As the flexure of the peripheral zone, will depend on the overall curvature of the cornea, it might be expected that some wearers will experience poor fitting [21] or an EPC, as outlined previously. Additionally, this change may be more pronounced for toric soft contact lenses due to the thickness changes and the influence of the stabilisation design.

**Fig 1:**
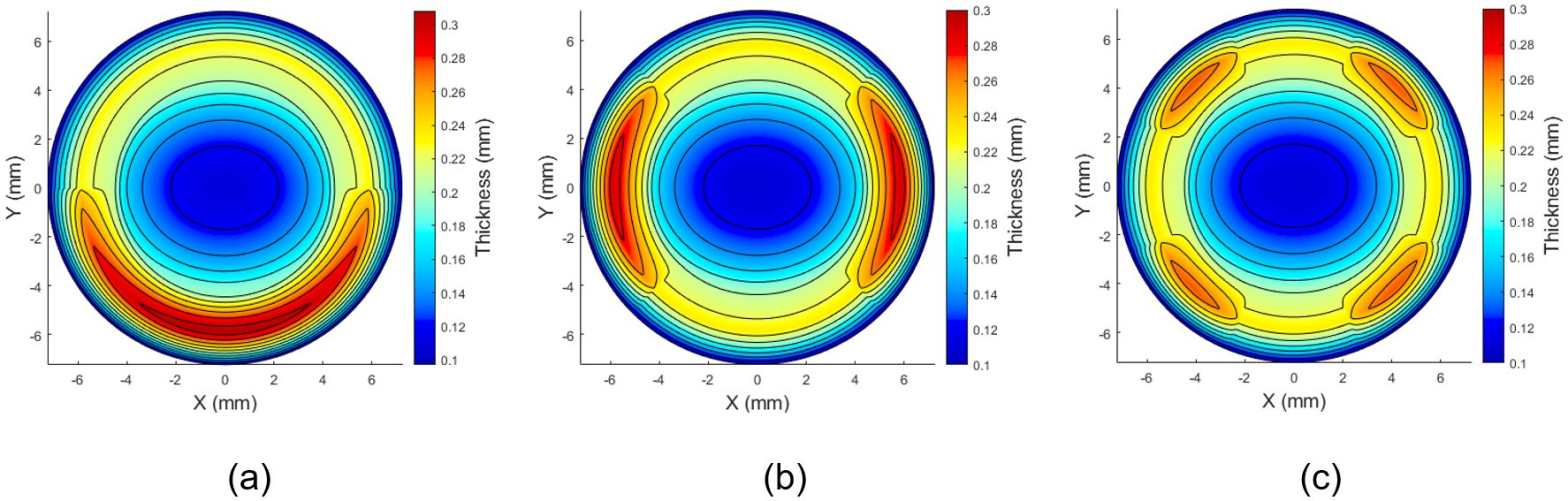
Three designs of a soft contact lens with a base curve 8.2 mm, diameter 14.5 mm, spherical power -2.0 DS, cylindrical power +1.0 DC at axis 90° and central thickness 0.15 mm: (a) Gravity-based single-prisms stabilised lenses (G1P), (b) Blink-based two-prisms stabilised lenses (B2P), (c) Blink-based four-prisms stabilised lenses (B4P).

**Fig 2:**
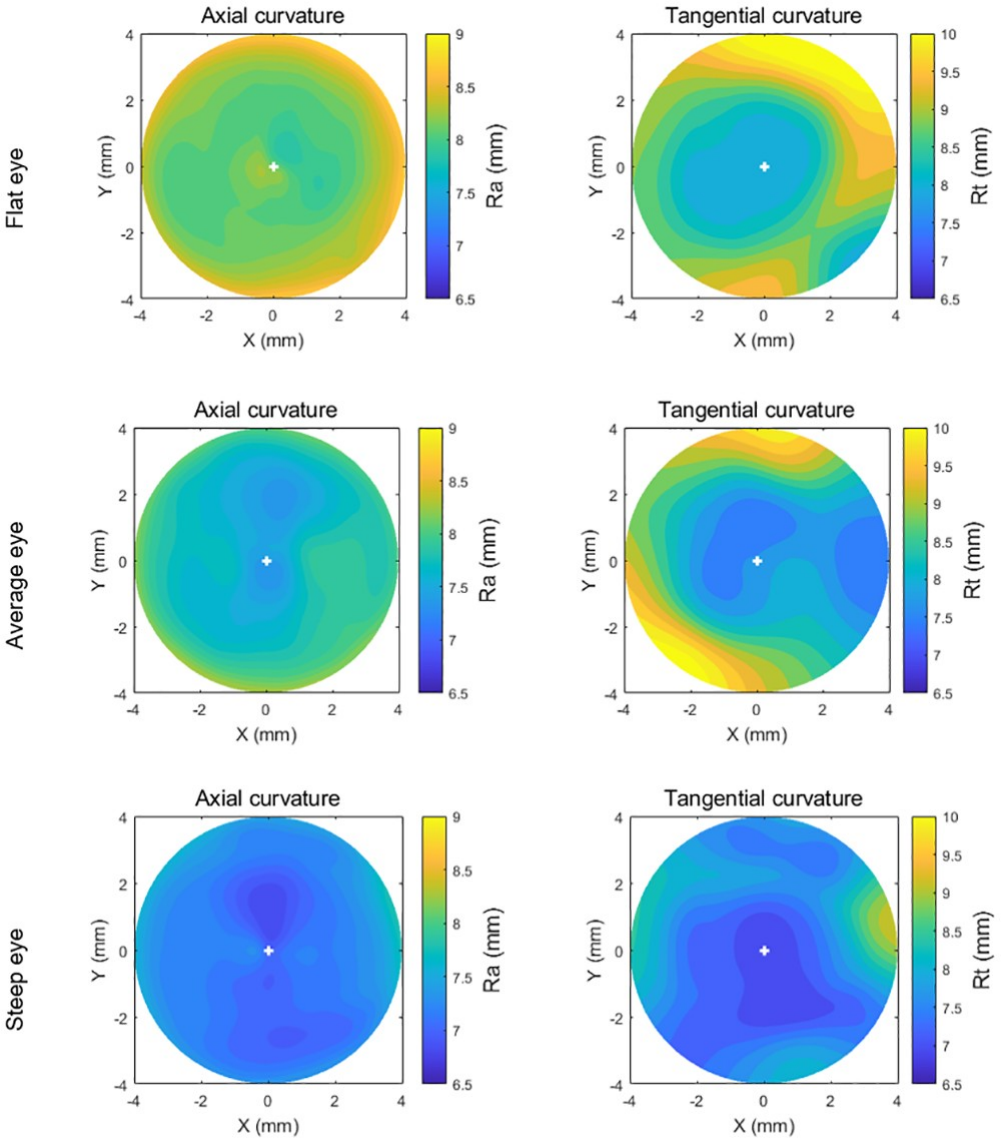
Axial and tangential curvature (Ra, Rt) maps of the flat, average, and steep eyes used in the current study.

The three most common soft toric stabilisation designs in the market are: (1) single prism (commonly known as prism ballast) or gravity-based stabilisation (G1P), Fig 1a; (2) two-prism blink-based stabilisation (B2P), Fig 1b; and (3) four-prism blink-based stabilisation (B4P), Fig 1c.

In addition to the main requirement of correcting refractive errors, soft contact lenses are designed to fit the eye. Although the design of the optic zone is restricted by the refractive power prescription, the design of the periphery zone has been left to the soft contact lens designer to construct using experience and common sense. There has been comparatively little work performed on peripheral zone design when compared to the research which has been conducted on the optic zone [22, 23].

The contact lens’s capacity to refract light (refractive power) is a function of its surface shape and its refractive index which is constant for any material. As a soft contact lens settles on the eye, it changes its surface curvature as a result of being subject to the eyelid pressure and the tear surface tension force. For this reason, the soft contact lens’s refractive power on-eye is not the same as it is off-eye, where the lens is designed, manufactured and passed its quality control final inspection. The design of the peripheral zone affects the flexure of the whole soft contact lens and hence, affects the EPC within the optic zone. Thus, the results of this study explain unexpected requirements for over-refraction for certain subjects seen in clinical practice, depending on their corneal shape.

The first peripheral zone design investigated in this study was the G1P, which is widely used in the contact lens industry and comprises a thicker section at the bottom of the contact lens. This design works with gravity to stabilise the lens automatically since the lower half is thicker, and therefore heavier, than the rest of the lens. The G1P design assumes that the weight of the thicker portion reacts with gravity such that when the centre of gravity is directly beneath the centre of rotation, the moment is reduced to zero, and so the lens stabilises in this position [24]. This type of stabilisation has minimal eyelid interaction and has the disadvantage of causing lens mis-location if the head is moved from the vertical.

B2P designs evolved from the early forms of “dynamic stabilisation” where the upper and lower portions of the lens were thinned to create a horizontal band of thicker material which interacted with the eyelids [25]. This design evolved into specific zones at 3 and 9 o’clock positions which interact with the lids in a more controlled fashion.

B4P designs [26] replace each prism zone with two smaller ones placed above and below the horizontal plane in order to interact with the eyelids more directly [27] and have advantages over other toric designs under a range of viewing conditions [28]. To gain a tangible perspective of how the three lenses change when placed on eyes, they needed to be tested on a range of different shaped corneas. The optimal soft contact lens adaptation, which allows patients’ comfort, good quality of vision and minimal interference with ocular surface functions and metabolism, is the result of a delicate balance between eye and lens dimensions and mechanical properties.

In order to simulate different contact lens fitting scenarios, three sets of eyes and contact lens fits were modelled by considering flat, average and steep corneas, the selection of eyes was carried out considering Gilani’s population study [29], where the median of the flat power simulated keratometry (Sim-K) was 43.8 D, and the bounds of “flat” and “steep” corneas were determined by applying one standard deviation of ±1.38 D. Both axial and tangential curvature maps of the three eyes used in this study are presented in Fig 2. Additionally, the trigonometries behind these maps are given in Appendix A.

This study investigates the effect of employing three different peripheral zone stabilisation designs on the EPC then determines which feature influences on-eye EPC, the eye shape or the contact lens design. Although the authors’ previous studies have been conducted on the effect of contact lens power on EPC [5, 30], this work introduces a new and more relevant approach, where the eye is not considered as a rigid solid body. In terms of design, the lenses considered in this study are designed using three peripheral zone designs, whereas the previous studies [5, 30] only considered a single peripheral zone. Previous studies [5, 30] employed lenses that, despite being valid designs, do not match those that are used in the soft contact lenses industry. The current work utilises lenses with a reduced concave geometry, thus allowing for improved tear circulation and for better compatibility with those used in the industry.

## Materials and methods

### Participants

The presented record review used fully anonymised secondary data, which according to the University of Liverpool’s Policy on Research Ethics, ethical approval was unnecessary. Nevertheless, the study followed the tenets of the Helsinki Declaration of 1964 (revised in 2013). Written informed consent was also provided in the primary data collection by all participants for the use of their de-identified data in scientific research [31].

In order to represent the normal corneal shape range, three sets of corneal topographies obtained using an Eye Surface Profiler (ESP) (Eaglet Eye BV, Houten, The Netherlands) from three subjects were selected for processing in this study. These were classified as flat, average and steep corneas [32] in terms of their flat simulated keratometry readings (flat-K): the average eye was within 0.5 SD of the average flat-K, the flat and the steep eyes were below or above 1.5 SD [29]. The “flat” and “steep” corneal shapes were taken from the right eyes of 28 and 25-year old females respectively (Flat Sim-K = 41.8 D & 46.8 D, Astigmatism = -0.17 D & -1.9 D, Angle = 2° & 3°). The “average” corneal shape was taken from the left eye of a 24-year old male (Flat Sim-K = 43.8 D, Astigmatism = -1.7 D, Angle = 3°). The selection of suitable eyes for inclusion in the study was carried out based on the corneal topography population study of Gilani [29]. Accordingly, the median of the “average” Sim-K was set to 43.8 D, and the bounds of flat and steep corneas were determined by applying one standard deviation of ±1.38 D [29]. By applying this classification to the patient data set, the corneas were classified as “flat” if the flat meridian power was less than or equal to 42.4 D, “steep” for flat meridian power of 45.2 D or above, and “average” if it was in-between. Recorded data was reviewed and three eyes from healthy participants were selected for this study, based on their geometry.

### Data processing

The eyes’ surface data was exported from the ESP software in the form of MATLAB (MathWorks, Natick, USA) binary data container format (*.mat) where the characteristics of eyes, as measured by the ESP system were extracted. Each selected eye’s topography data was processed by custom-built MATLAB codes completely independent from the built-in ESP software digital signal processing algorithms. Each eye’s topography data was measured over more than 250,000 points on average. The ESP data comprises the anterior front-surface of the eye up to 20 mm without extrapolation. The Z-axis represents the axial direction of the eye with an origin point resting on the corneal apex with the eye on the negative side. In order to determine the cornea’s asphericities, conic models were fitted to each cornea’s anterior surface of the flat, average and steep eyes where asphericity *q* and corresponding shape factor *k* = *q* + 1 were obtained. The asphericity factor *q* is synonymous to the overall curvature of the cornea, with positive values leading to increased steepness and negative values inducing a flattening effect [33, 34]. Fitting was carried out by minimising the mean squares error between the corneal surface and the fitted conic model for each eye. The corneal models considered in this study utilised radii of 7.45, 7.04 & 6.42 mm, asphericities of *q* = -1.2, -1.107 & -1.154 and corresponding shape factors k = -0.2, -0.107 & -0.154 for the flat, average and steep eyes respectively.

The scleral portion of the measured eye’s topography data was separated from the corneal portion by detecting the limbus position. The detected limbal position on the eye’s anterior surface was then identified through the three-dimensional (3D) non-parametric method introduced by Abass et al. in 2018 [35]. The limbus detection algorithm was based on the fact that the cornea and the sclera have different curvatures [36] and the limbus is the boundary where the corneal curvature changes to match that of the scleral globular shape [37]. Therefore, the position of the limbus was detected by locating the turning point of the raw elevation 2^nd^ derivative at each meridian.

When the limbus was detected; the scleral topography data was first processed through an edge-effect elimination process where topographical artefacts caused by the eyelash’s interference or tear pooling were removed using the technique introduced by Abass et al. [31]. Once the scleral topographical data is cleared of measurement artefacts, it was then fitted to a sphere using the least squares error method, minimising the fitting error *Err* for every point i of the n points as described in Eq 1

$$Err=\overset{n}{\underset{i=1}{\sum }}{\left({\left(X_{i}-X_{c}\right)}^{2}+{\left(Y_{i}-Y_{c}\right)}^{2}+{\left(Z_{i}-Z_{c}\right)}^{2}-R_{s}{}^{2}\right)}^{2}$$ (1)

Where X_i_, Y_i_ and Z_i_ are the scleral height data, X_c_, Y_c_ and Z_c_ are the best-fitted sphere’s centre coordinates, and R_s_ is the radius of the sclera.

To construct a 3D eye geometrical model without losing the precision of the measured part of the sclera, the measured portion of the anterior sclera was used in the construction process while the best-fitted spherical surface was only used in the areas where no scleral surface measurements were available. The constructed eye geometry was then used to build the finite element model as will be shown later in this study.

#### Contact lens design

The back and front-surfaces of soft contact lenses modelled in this study were configured to approximate those that are commercially available. However, simulation of the specific design of particular brands was not possible, as this is protected non-available information. Despite this, the precise engineering principles of soft contact lens design were carefully considered in this study.

**Fig 3:**
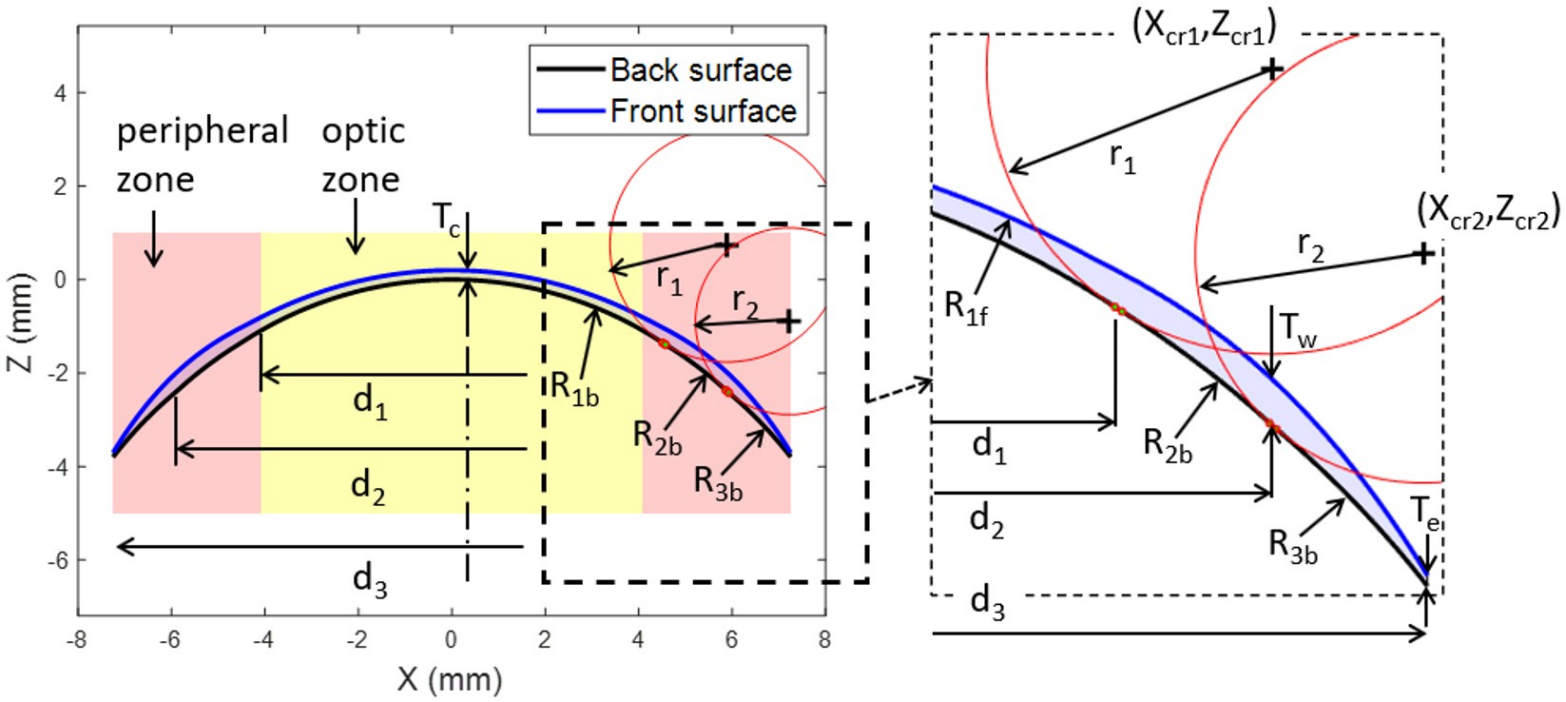
Geometry parameters of a blink-based two-prisms stabilised lens (B2P) with base curve 8.2 mm, spherical power -2.0 DS, optical cylindrical power -1.0 DC, axis 90° and central thickness 0.2 mm. In this figure, fillets radii r_1_ and r_2_ were set to 2.5 mm and = 2.0 mm respectively. See Appendix B for more details.

Each lens surface was divided into an optical zone and a peripheral zone (Fig 3). The front-surface was designed to achieve three main requirements; the necessary optical power within the optic zone of the lens, stabilisation through a balance prism profile (T_w_) and a specified edge thickness (T_e_) at the point where the peripheral zone merges into the edge profile.

Finally, due to clinical considerations, practical reasons and manufacturing restrictions, lenses cannot be designed with sharp edges when two zones meet. To mitigate this, any sharp edges must be smoothed. This study presents the mathematical details of how the geometry and locations of these fillets were determined.

A custom-built MATLAB script was written to generate the geometrical shape of the lenses based on the type of balance zone design, the design parameters and the optical power values. Although the design process has been covered in previous studies [5, 30], for completeness, details of the lens design are presented in Appendix B. In addition to the details dealt with in previous work, the fillet design and new peripheral zone design techniques are also outlined (see S1 File).

#### Finite element modelling

In this study, soft contact lens models were fitted on three eye models representing flat, average and steep profiles, for a total number of 567 FE models with an average central processing unit (CPU) time of 12.2 min per model while a 4-core processor HP tower workstation (HP Inc UK Limited, Reading, UK) was being used. FE overall simulation wall-clock time was estimated as 117.3 hrs with an average of 12.4 min per model. Sets of lenses’ geometrical profiles were digitally generated through the MATLAB software for the three investigated designs G1P, B2P and B4P before being further processed to build an FE model for each lens. The cylindrical lens set was designed with 21 nominal powers ranging from -10 DC to 10 DC at cylinder axes 90°, 45°, and 0° in one dioptre optical power step.

As the ESP can only measure the anterior surface of the eye and is not capable of measuring the posterior cornea, the central corneal thickness (CCT) was taken as its reported average value of 0.55 mm [38] then increased to 0.70 mm and 0.56 mm at the peripheral corneal zone and equatorial ring respectively [39, 40]. Additionally, at the posterior pole, the thickness was taken as 0.84 mm [41]. Ocular globe wall thickness was varied linearly with the elevation angle among the previously mentioned regions. Eight-node first-order continuum solid hybrid brick elements ‘C3D8H’ were used in one layer of elements to build the eye model and 2 layers to build the contact lens models in ABAQUS (Dassault Systèmes, Vélizy-Villacoublay, France) FE software package licenced to the University of Liverpool, UK. Normally, the in-vivo human eye globe topography is measured whilst the eye is stressed due to the intraocular pressure (IOP) hence, cannot be used for modelling without pre-processing. To achieve the eyes’ stress-free geometry (at IOP = 0 mmHg), eye globe models were initially built with the inflated dimensions, then a stress-free adaptation of each model was calculated individually by following the iterative method presented in [42]. In each case, the stress-free model was computed by considering an average IOP of 15 mmHg [43] and a maximal node position error less than 10^−4^ mm. Once the stress-free models were determined, they were pressurised to IOP = 15 mmHg through a uniformly distributed static pressure on the internal surfaces of the eye globe model to mimic the aqueous and vitreous behaviour. The ABAQUS nonlinear geometry option “NLGEOM” was set to “ON” during the inflation process and subsequent steps. This option allows loads to be applied incrementally, whilst updating the stiffness matrix for each increment. Hence ABAQUS allows nonlinear materials to be used for certain parts without altering linear FE formulation for linear materials of other parts of the model.

The FE mesh convergence study of eye’s model was carried out through applying internal pressure of 15 mmHg on the internal surface of 14 eye models, half of them are double-layered, then monitoring the relevant anterior eye’s apex displacement. Single layers models were constructed using 804, 5004, 20004, 80004, 180004, 320004 and 500004 nodes then inflated. Relevant apex displacement in single-layer models were reduced by 0.0%, 3.0%, 3.8%, 4.7%, 4.7%, 4.7% and 4.7% respectively while the 804-node model was taken as the datum. Double layers models were constructed using 1206, 7506, 30006, 120006, 270006, 480006 and 750006 nodes where apex displacement was found to be reduced by 0.0%, 3.2%, 3.9%, 4.7%, 4.8%, 4.9% and -5.0% respectively while the 1206-node model was taken as the datum. The outcomes showed that a number of the elements equal to 40000, arranged in 200 rings (80004 nodes), in a single layer converged to the displacement of 201.545 μm at the apex node and was selected as an optimal number of elements for this simulation as it compromised between the computational resources and the accuracy of the solution.

The contact lenses mesh was tested by 10 Plano lenses models, five of them were single-layered with 20166, 20526, 21846, 23966 and 26886 nodes and the other five models were double-layered with 20247, 20787, 22767, 25947 and 30327 nodes respectively. All contact lenses models were tested when being fitted to the selected 80004 node eye model while the lenses apex displacement was recorded. Lenses apex displacement was reduced by 0.0%, 0.8%, 1.0%, 1.1% and 1.2% in single-layered models and by 0.0%, 0.8%, 1.0%, 1.0% and 1.0% in double-layered models respectively.

The outcome demonstrated that models with the number of the elements equal to 11680 arranged in 20 rings (22767 nodes) in double layers converged to the displacement of 203.425 μm at the apex node and was selected as the optimal number of elements for the lens in this simulation. Lenses were designed with an optic zone diameter d_1_ = 8 mm, balance zone diameter d_2_ = 11.25 mm, overall diameter d_3_ = 14.5 mm, and base curve R_1b_ = 8.2 mm.

**Fig 4:**
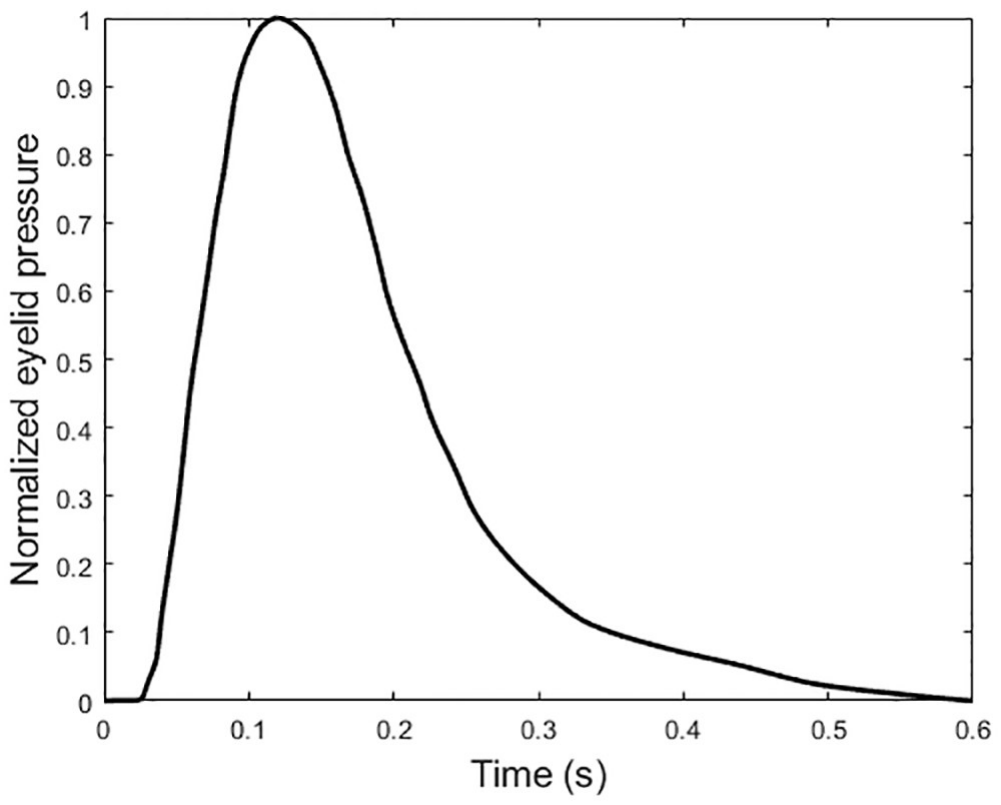
Normalised eyelid pressure magnitude with time. This distribution is based on the palpebral aperture measurement as reported in [34].

The contact lens material was simulated with the properties of non-ionic hydrogel with 77% water content (Contamac, Saffron Walden, England, UK) and the eyelid effect by a nonlinear dynamic upper eyelid blink pressure of P_1_ = 8.0 mmHg [44] applied dynamically to the front surface of the lens. This application occurred 0.6 s after applying the IOP pressure in a normalised amplitude following Kwon’s high-speed camera characterisation of the blinking kinematics [45], Fig 4. The effect of the tear layer was simulated by applying the surface tension of tear fluid of P_2_ = 43.6 mPa [27] to the back surface of the contact lens. Using the FE software ABAQUS, the full FE model consisted of two parts, the contact lens and the eye with a single interface between them. Material models are detailed in Appendix C.

In the context of ABAQUS FE models, the anterior corneal surface and contact lens back-surface were taken as master and slave surfaces respectively. The interaction between these surfaces was further defined using a coefficient of friction of 0.01 [46]**.**

**Fig 5:**
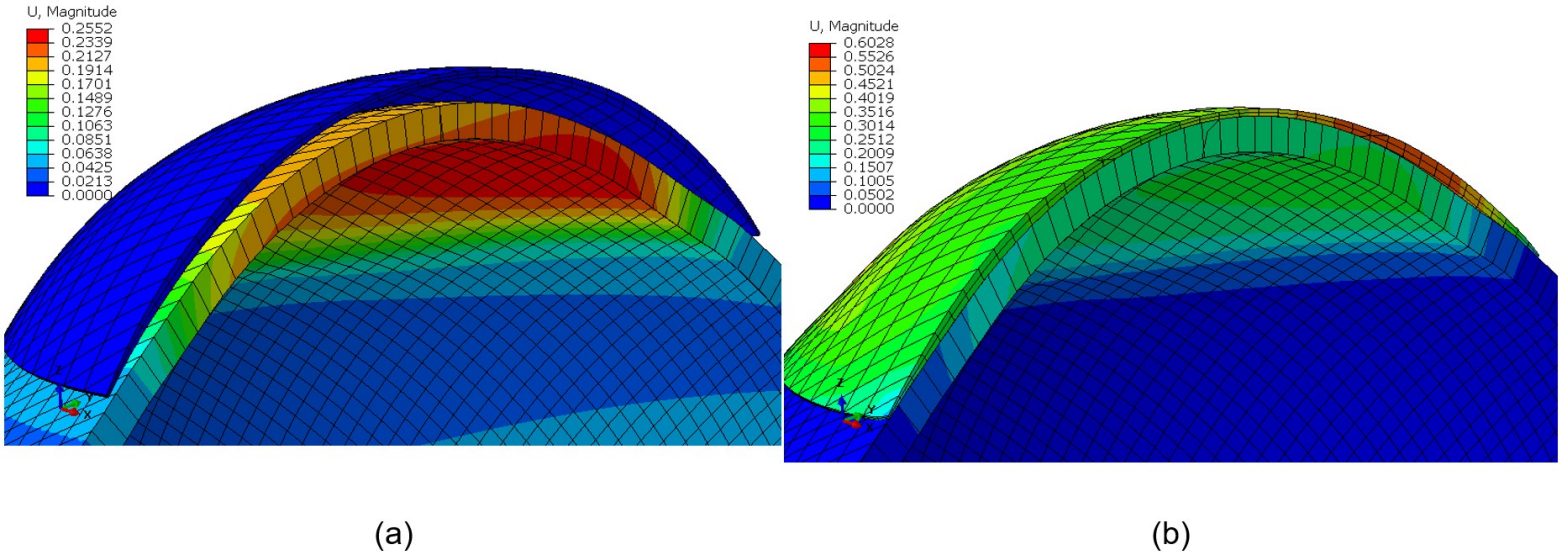
Contact lens finite element model for a G1P lens design fitted to an average eye (IOP = 15 mmHg) on the ABAQUS finite element software: (a) before fitting, (b) after fitting. Colour scale is representing the magnitude of the displacement (U) in mm. Eye’s equatorial nodes were constrained in axial directions.

The displacement of the eye’s equatorial nodes was constrained in the Z-direction, and both the corneal apex and posterior pole nodes were constrained in the X-direction and Y-direction. The lens, however, was constrained by preventing X and Y displacement at the optical centre, Table 1 & Fig 5.

**Table 1:** Finite element simulation parameters.

Step	Description	Integration scheme	Loading condition	Time
1	Stress-free iterations [42]	Implicit	Static	Normalised increments (0:1)
2	Inflation, IOP = 15 mmHg [43]	Implicit	Static	Normalised increments (0:1)
3	Eyelid pressure 8.0 mmHg [44]	Implicit	Dynamic	0.6 s, see Fig 5 [45]
4	Surface tension 43.6 mPa [27]	Implicit	Static	Normalised increments (0:1)

Once the design phase was complete, and the dimensions of lenses were obtained, the volume of the lenses’ peripheral zones, which contains the stabilisation prisms, were calculated as a ratio (V_p_) of the total lens volume via the MATLAB “boundary” function. This value indicates how much material was put in the peripheral zone compared to the overall material content of the contact lens.

#### Light raytracing

**Fig 6:**
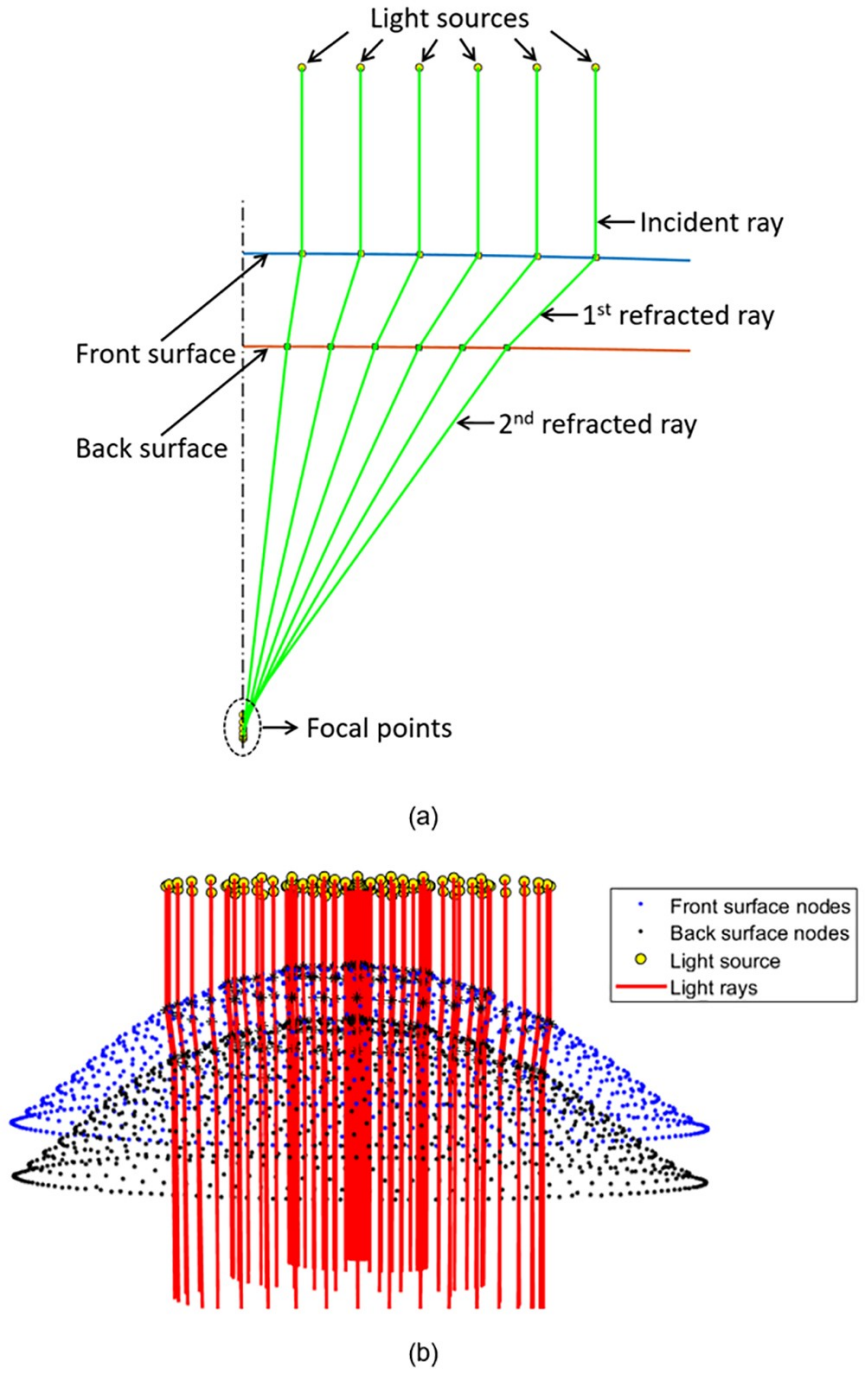
Light raytracing according to Snell’s law in (a) a single meridian, (b) three-dimensional analysis (lens’s thickness has been increased in this figure for displaying purposes).

To measure the EPC incurred by the conformance of each soft contact lens to the cornea, the light raytracing technique outlined in [30] was employed. A custom-built MATLAB script performing light raytracing across the lens optic zone was written and validated using the AutoCAD software^®^ (Autodesk, Inc., San Rafael, California, USA). This technique allows for the simulation of a large number of light-rays as they travel through the lens, Fig 6b. Prior to the ray-tracing analysis, the coordinates of the FE models, pre and post conformance, were exported and fitted to surfaces using piecewise cubic interpolation. The direction of each light-ray before, during and after entering the lens, was then deduced through the use of Snell’s law [47].

**Fig 7:**
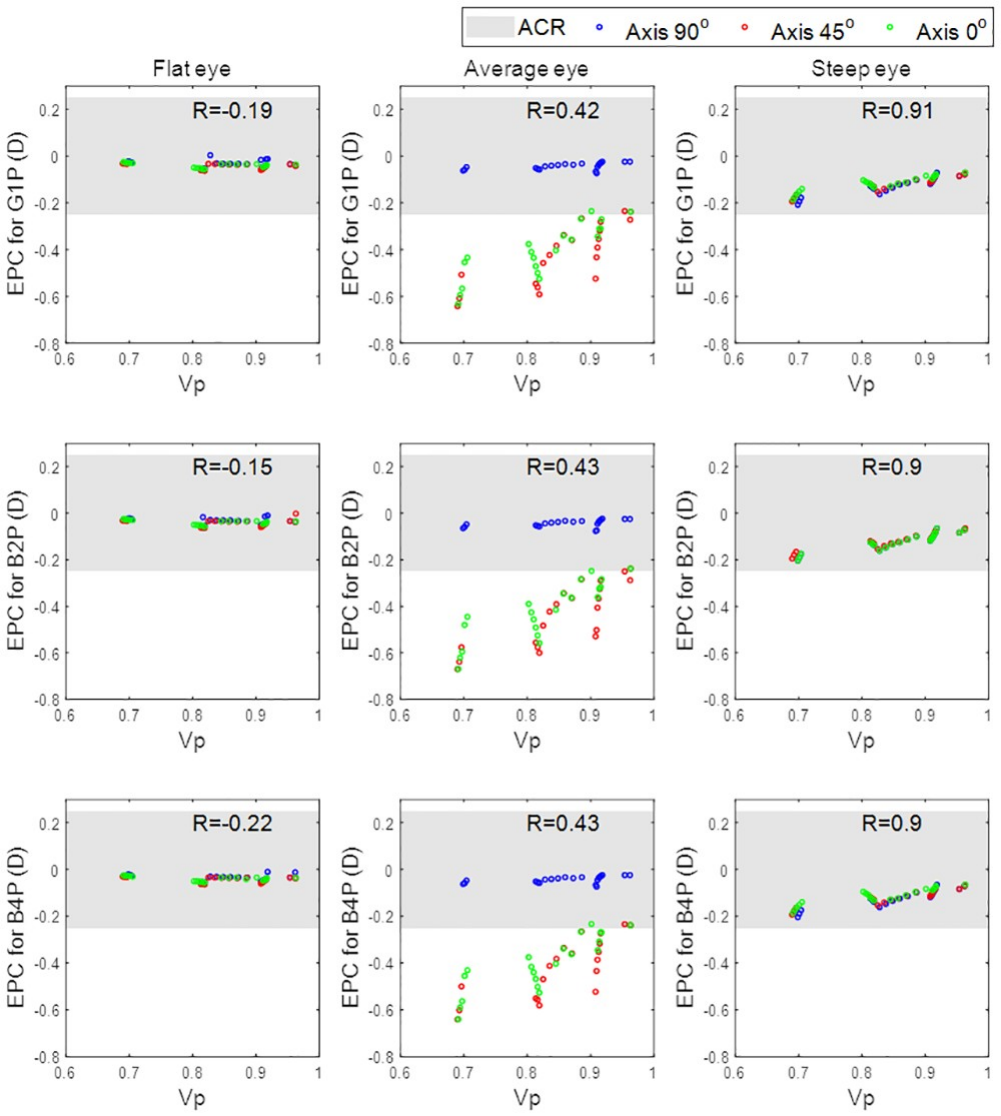
Effective power change (EPC) for cylindrical lenses with axis 90° as a function of the peripheral zone volume (V_p_) when fitted to the flat eye (1^st^ column), average eye (2^nd^ column), and steep eye (3^rd^ column). The three investigated designs are plotted in rows.

The focal point was then identified by finding the average location at which the light-rays intersect the optical axis, Fig 7a. The distance between this point and the lens apex was then calculated to yield the focal length, *f*. When inverted, the focal length can be used to produce a value for the lens’s optical power. The difference in optical power produced by the lens after and prior to conformance was taken as the effective power change, EPC. The validated light raytracing script was run for each of the considered contact lens geometries, before and after fitting to the three corneal geometries. This allowed for the identification of the EPCs and their standard deviations across the lens’s optic zone.

Acceptance criteria range (ACR) for the level of EPC that would initiate a clinically significant response was set at ±0.25 DC for practical reasons, as this reflects the minimum change in power used in clinical optometric refractions.

### Statistical analysis

The statistical analysis carried out on the results of this study was performed using the Statistics and Machine Learning Toolbox of the MATLAB software. The null hypothesis, at 95.0% confidence level testing, was used to investigate the inferences of the findings based on statistical evidence. The normal distribution of the samples was confirmed using the Kolmogorov-Smirnov test via MATLAB [48] then the two-sample t-test was applied to investigate whether there was a significance between pairs of data sets and to confirm whether the assessed findings represent an independent record. The probability value (p) is an element in the closed period 0.0 to 1.0 where values of p higher than 0.05 indicate the validity of the null hypothesis [49]. The MATLAB function ‘ttest2’ was used and the returned p-value in addition to binary test decision for the null hypothesis. The correlation coefficient used in this study (R) is a measure of the linear dependence of two variables [50]. R values below 0.3 were considered as an indication of weak correlations; R values in the range 0.3 to 0.7 were considered as an indication of moderate correlations; and finally, R values above 0.7 were considered as an indication of strong correlations [51].

## Results

**Fig 8:**
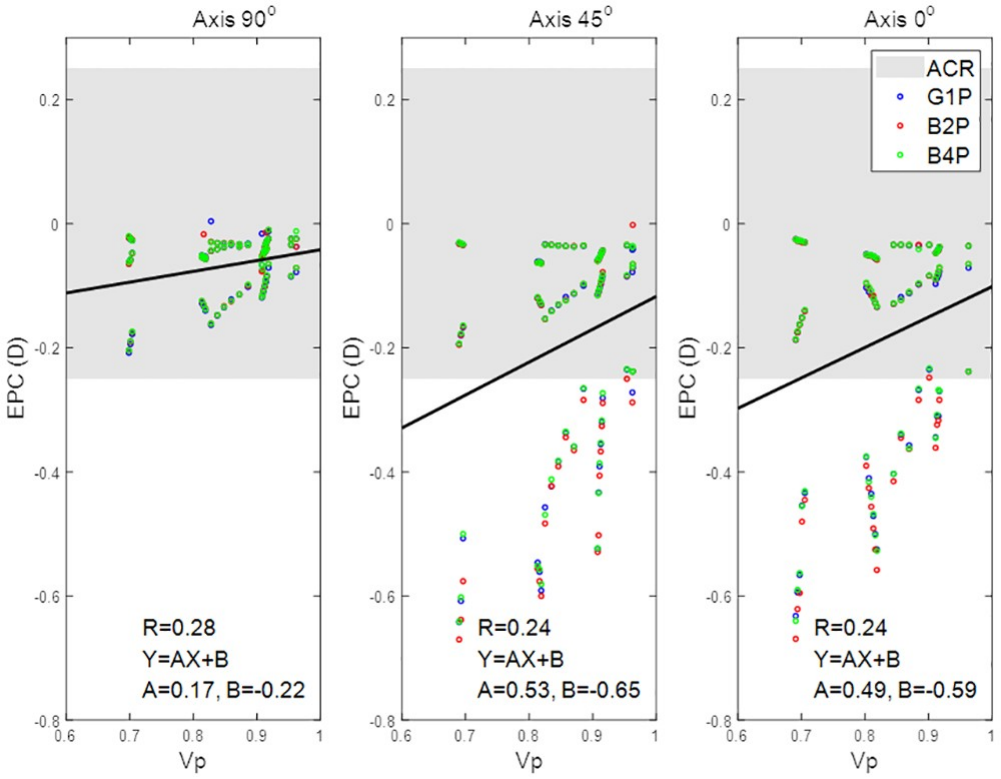
Effective power change for lenses with axis 90°, 45°, and 0° as a function of the peripheral zone volume.

When evaluating the flat eye models, the correlation between effective power change, EPC, and peripheral zone volume, V_p_, was weak, Fig 8. This was evident in all three of the investigated peripheral zone designs (G1P, B2P and B4P) where the correlation coefficients were -0.19, -0.15 and -0.22 respectively. The correlations were, however, moderate in the average eye models (R = 0.42, 0.43 and 0.43 respectively) and strong in the steep eye models (R = 0.91, 0.9 and 0.9 respectively).

When the effect of the toric axis on the EPC was investigated for the three eyes, a proportional relationship was found, Fig 8. Moreover, the correlation between V_p_ and EPC decreased slightly from 0.28 with an axis at 90° to 0.24 with the axis at 45° and the axis at 0°. Changing the toric lens axis away from 90° induced a negative EPC. Additionally, investigated lenses with axes of 45° and 0° generally recorded EPCs outside the ACR.

**Fig 9:**
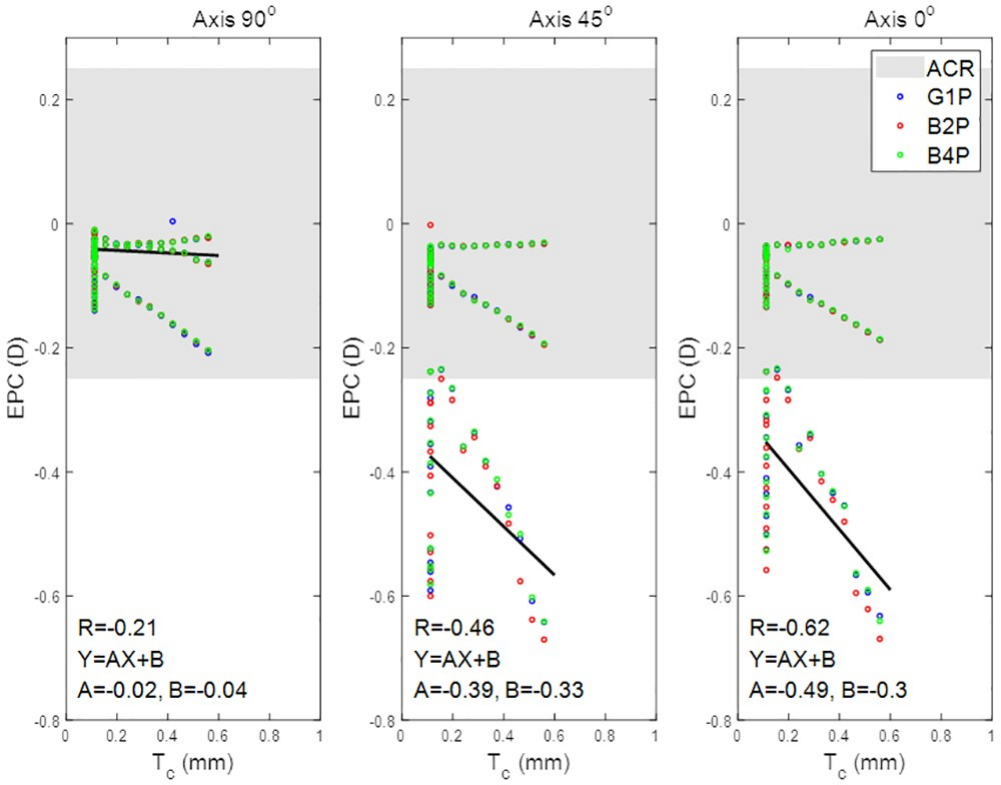
Effective power change for lenses with axis 90°, 45°, and 0° as a function of the lens’s central thickness.

When the effect of the lens’s central thickness T_c_ was explored, it was clear that, for positive nominal powers (up to 10 DC), the effect of T_c_ on the EPC was counter to the effect of Vp and the inverse correlations were found, R = -0.21, -0.46 and -0.62 for axis 90°, 45°, and 0° respectively. T_c_ recorded no correlation with the EPC within the negative nominal power ranges (down to -10 DC). Both axes 45° and 0° cylindrical lenses recorded EPC below the ACR in most of the nominal cylindrical power range, Fig 9. No significant differences were recorded among the three designs G1P, B2P and B4P designed with axes of 90°, 45° and 0°.

## Discussion

The study investigated the impact of different prism designs in terms of the EPC against the nominal power of the contact lenses. Although the results showed that all of the investigated designs were changing their power as a result of altering their shape on the eye, all three recorded EPC within the ±0.25 DC range when fitted to the three eyes (flat, average, steep). When the influence of the peripheral zone was investigated, it was clear that the V_p_ was more strongly correlated to the steep (R≅0.9) and the average eyes (R≅0.43) than the flat eye (R≅0.26) regardless of the choice of peripheral zone design. However, when the three eyes were fitted with cylindrical lenses with a different axis, the correlation between the V_p_ and the EPC slightly decreased from 0.28 to 0.24 as the axis was reduced from 90° to 0°.

Unlike in previous studies [5, 30] where the eye was treated as an elastic rigid body, the hyper-elastic material properties and dynamics of the eye were considered through updating the modelling process. This has been achieved by modelling the eye’s material in four regions (cornea, anterior sclera, intermediate sclera, and posterior sclera) with hyper-elastic Ogden models. This update also allowed consideration of the whole eye geometry precisely instead of the anterior portion only, considering the existence of the intraocular pressure (IOP) and applying the eyelid pressure dynamically as a function of time [45] instead of considering it as a static load [5, 30].

In all investigated cases, the eye shape had more influence on the EPC than the peripheral zone design. However, and not as previously reported [52], the volume of the peripheral zone was highly correlated with the EPC in the moderate and steep eye population. Additionally, the central thickness was only correlated with EPC in lenses with positive nominal power.

It was not clear that anterior eye shape influences on-eye EPC however, as the contact lens’s design has a limited effect of the on-eye refractive performance. Moreover, using the lens’s central thickness as an estimator for calculating EPC [52] is not possible with negative powered lenses, therefore, the lens’s peripheral zone ratio of the total lens volume could be used as a linear estimator for EPC. However, the relationship between the V_p_ and the EPC is dependent on the eye shape.

In this study, the simulation of the soft contact lens performance on the eye was limited to observing the deformation of the lens and the associated EPC on each corneal shape in response to applied eyelid pressure. Increasing the number of eyes could strengthen statistical conclusions, however, the three eyes used in this study were carefully selected from a database containing 125 pairs of eyes to represent good examples of flat, average, and steep eyes and allow the study to vary contact lenses’ design with a practical number of models (567 models). In this study, the effect of the tear layer was simplified and simulated by applying the surface tension of the tear fluid of 43.6 mPa [27] to the back surface of the contact lens as no fluid-structure interaction analysis has been carried out in this study.

The rotation and translation of the soft contact lenses were not considered in the models used in this study to simplify the convergence towards stable solutions in the ABAQUS FE software. It was not possible to simulate accurate rotational effects of the contact lens as the current model does not calculate extraneous factors such as tear volume, eyelid position and movement characteristics or gravitational effects. Despite this, this study has highlighted the dependence of EPC on the corneal geometry and demonstrated that, although specific designs can be used to reduce EPC, the corneal geometry will have an overarching effect.

## Appendix A: Axial and tangential radii of curvature

The least squares error method was applied to fit the best circle to each meridian and the radius of each fitted circle was used as a radius of curvature for this meridian. Local axial and tangential curvatures were calculated for 359 meridians with a 1.0° angular step covering the whole measured area of the cornea up to *x* = 4 mm radius.

**Fig 10:**
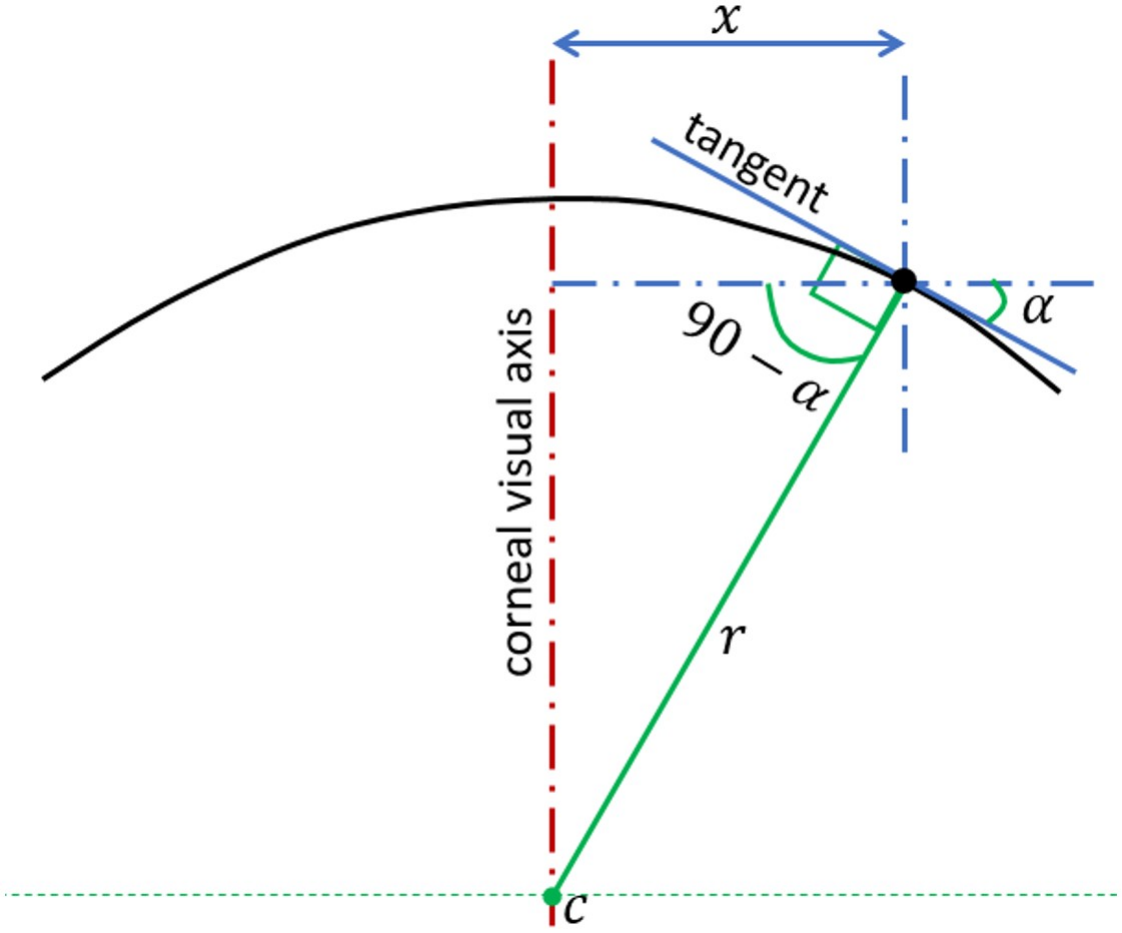
Determination of corneal surface axial radius of curvature (r) at a certain meridian plane. In this method, the centre of the curvature (c) is always restricted to the corneal visual axis.

**Fig 11:**
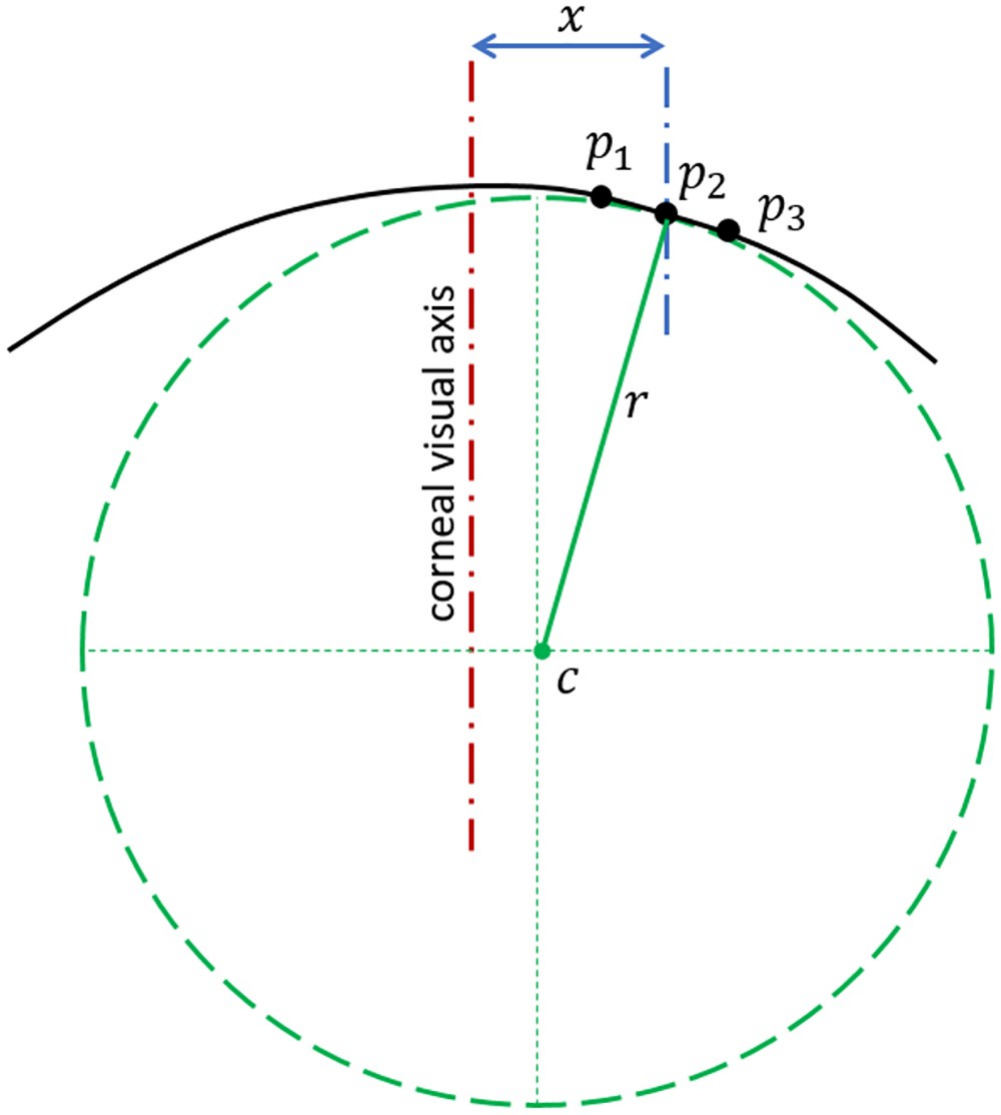
Determination of corneal surface tangential radius of curvature (r) at a certain meridian plane. In this method, the centre of the curvature (c) is not restricted to the corneal visual axis.

While centres of axial curvatures were assumed to lie on the corneal visual axis (Fig 10), the centres of tangential curvature were free to be at any position but still within the relevant meridian plane (Fig 11). [53] As illustrated in Fig 10, the axial radius of curvature at any point is calculated as:

$$r=\frac{x}{\text{cos}(90-\alpha )}$$ (2)

where *α* is the tangent angle at this point. On the other hand, the tangential radius of curvature at any point, *p*_2_, on the corneal surface can be calculated by fitting a circle to the three consecutive points *p*_1_ (*x*_1_, *y*_1_, *z*_1_), *p*_2_ (*x*_2_, *y*_2_, *z*_2_), *p*_3_ (*x*_3_, *y*_3_, *z*_3_) along the relevant meridian resulting in the value:

$$r=\frac{1}{2}\frac{\left\|p_{1}-p_{2}\|\|p_{2}-p_{3}\|\|p_{3}-p_{1}\right\|}{\left(p_{1}-p_{2})\times (p_{2}-p_{3}\right)}$$ (3)

as illustrated in Fig 11.

Both axial and tangential curvature map estimation methods handling local segments of each corneal meridian as perfect circles, however, the method used for the axial curvature map calculation restricts the surface curvature centres to the corneal visual axis.

## Appendix B: Soft contact lens design

### Back-surface design

Geometrical parameters considered in the design of the lens back-surface include the back optic zone radius or the base curve (R_1b_), the back transient zone radius (R_2b_), the peripheral curve radius (R_3b_) and the overall lens diameter (d_3_), Fig 3. As outlined in a previous study [5], X and Z coordinates (X_c_, Z_c_) of the centres of radii R_1b_, R_2b_ and R_3b_ were calculated as:

$$\begin{array}{cc} {\text{X}}_{\text{c}1}=0, & {\text{Z}}_{\text{c}1}=-{\text{R}}_{1\text{b}} \end{array}$$ (4)

$$\begin{array}{cc} {\text{X}}_{\text{c}2}=0, & {\text{Z}}_{\text{c}2}={\text{Z}}_{\text{c}1}-{\text{R}}_{2\text{b}}\text{cos}({\text{sin}}^{-1}\frac{{\text{d}}_{1}}{2{\text{R}}_{2\text{b}}})+{\text{R}}_{1\text{b}}\text{cos}({\text{sin}}^{-1}\frac{{\text{d}}_{1}}{2{\text{R}}_{1\text{b}}}) \end{array}$$ (5)

$$\begin{array}{cc} {\text{X}}_{\text{c}3}=0, & {\text{Z}}_{\text{c}3}={\text{Z}}_{\text{c}2}-{\text{R}}_{3\text{b}}\text{cos}({\text{sin}}^{-1}\frac{{\text{d}}_{2}}{2{\text{R}}_{3\text{b}}})+{\text{R}}_{2\text{b}}\text{cos}({\text{sin}}^{-1}\frac{{\text{d}}_{2}}{2{\text{R}}_{2\text{b}}}) \end{array}$$ (6)

and the back-surface elevation, Z_b_ was constructed as

$${\text{Z}}_{\text{b}}=\{\begin{array}{ccc} {\text{Z}}_{\text{c}1}+\sqrt{{\text{R}}_{1\text{b}}{}^{2}-{\left(\text{X}-{\text{X}}_{\text{c}1}\right)}^{2}} & , & 0\leq \text{X}<\frac{{\text{d}}_{1}}{2}-x_{p1}\\ {\text{Z}}_{\text{c}2}+\sqrt{{\text{R}}_{2\text{b}}{}^{2}-{\left(\text{X}-{\text{X}}_{\text{c}2}\right)}^{2}} & , & \frac{{\text{d}}_{1}}{2}+x_{p2}\leq \text{X}<\frac{{\text{d}}_{2}}{2}-x_{p3}\\ {\text{Z}}_{\text{c}3}+\sqrt{{\text{R}}_{3\text{b}}{}^{2}-{\left(\text{X}-{\text{X}}_{\text{c}3}\right)}^{2}} & , & \frac{{\text{d}}_{2}}{2}+x_{p4}\leq \text{X}\leq \frac{{\text{d}}_{3}}{2} \end{array}$$ (7)

### Front-surface design

A typical initial central lens thickness T_c_ = 0.11 mm was used in all cases before being updated during the design process (Fig 3). The lens material refractive index (n) was set to 1.334 to simulate hydrogel optical characteristics [5], however, the lens nominal power (P_i_) was varied according to the required optical power to be consistent with the Lens Makers Equation (Eq 8) which was solved for optic zone front-surface curve R_1f_ in Eq 9

$${\text{P}}_{\text{i}}=(\text{n}-1)(\frac{1}{{\text{R}}_{1\text{f}}}-\frac{1}{{\text{R}}_{1\text{b}}}+\frac{{\text{T}}_{\text{c}}\;(\text{n}-1)}{{\text{n}\;\text{R}}_{1\text{f}}{\;\text{R}}_{1\text{b}}})$$ (8),

[54, 55]

$${\text{R}}_{1\text{fi}}=\frac{{\text{T}}_{\text{c}}{\left(\text{n}-1\right)}^{2}+\text{n}(\text{n}-1){\text{R}}_{1\text{b}}}{{\text{nR}}_{1\text{b}}{\text{P}}_{\text{i}}+\text{n}(\text{n}-1)}$$ (9),

[5, 30]

Front-surfaces were designed with the lens shape factor (k) set to the empirically estimated value of 0.75 to eliminate the spherical aberration on the central vision zone, Therefore, the lens front-surface was shaped meridian by meridian as:

$${\text{Z}}_{\text{fi}}={\text{T}}_{\text{c}}-\frac{1}{\text{k}}({\text{R}}_{1\text{fi}}-\sqrt{{\text{R}}_{1\text{fi}}{}^{2}-{\text{kX}}^{2}})$$ (10)

Where the subscript (i) stands for the meridian number and therefore i equals 1, 2, 3, …, 360 corresponding respectively to meridian angles θ = 0°, 1°, 359° rotating around the Z-axis in the anti-clockwise direction.

### Fillet design

To avoid sharp edges on the back-surface, fillets with radii r_1_ = 2 mm and r_2_ = 1.5 mm were introduced to connect the sections of the back surface with curvature changes of R_1b_ to R_2b_, and R_2b_ to R_3b_ respectively, Fig 3. The centre of the r_1_ fillet ($X_{c_{r1}}$, $Z_{c_{r1}}$) was calculated by finding the point at which the two sections under consideration would intersect if their radii were reduced by the fillet radius, r_1_. When this problem was solved exactly, the following relations were achieved,

$$\begin{array}{cc} X_{c_{r1}}=\frac{Q_{1}\sqrt{Q_{2}Q_{3}}-Z_{c2}\sqrt{Q_{3}Q_{4}}-Q_{5}}{2Q_{6}}, & Z_{c_{r1}}=\;Z_{c1}+\sqrt{{\left(R_{1b}+r_{1}\right)}^{2}-{\left(X_{cr1}-X_{c1}\right)}^{2}} \end{array}$$ (11)

where *X*_*ci*_ and *Z*_*ci*_ denote the coordinates of the centre of radius *R*_*ib*_ and the variables denoted by *Q* are shape factors, detailed in Table 2. The start and end points of the fillet were then computed by finding the two locations where a circle with centre (*X*_*c*1_, *Z*_*c*1_) and radius r_1_, intersects the lens surface. The result of this is a start (*x*_*p*1_, *z*_*p*1_) and end point (*x*_*p*2_, *z*_*p*2_) for the fillet given by,

$$\begin{array}{cc} x_{p1}=\;\frac{Q_{7}Q_{8}-\;z_{cr1}Q_{8}-2Q_{10}}{2Q_{11}}, & z_{p1}=\;Z_{c1}+\;\sqrt{R_{1b}{}^{2}-{\left(x_{p1}-X_{c1}\right)}^{2}} \end{array}$$ (12)

$$\begin{array}{cc} x_{p2}=\;\frac{Q_{12}Q_{13}-Z_{cr1}Q_{13}-2Q_{15}}{2Q_{16}}, & z_{p2}=\;Z_{c2}+\sqrt{R_{2b}{}^{2}-{\left(x_{p2}-X_{c2}\right)}^{2}} \end{array}$$ (13)

Applying the same process to the second change in lens geometry (R_2b_ to R_3b_) yielded a fillet with centroid ($X_{c_{r2}}$, $Z_{c_{r2}}$) and start (*x*_*p*3_, *z*_*p*3_) and end points (*x*_*p*4_, *z*_*p*4_) given by,

$$\begin{array}{cc} X_{cr2}=\;\frac{Q_{17}\sqrt{Q_{18}Q_{19}}+Z_{c3}\sqrt{Q_{18}Q_{19}}-2Q_{20}}{2Q_{21}}, & Z_{cr2}=\;Z_{c2}+\sqrt{{\left(R_{2b}-r_{2}\right)}^{2}-{\left(X_{cr2}-X_{c2}\right)}^{2}} \end{array}$$ (14)

$$\begin{array}{cc} x_{p3}=\;\frac{Q_{22}\sqrt{\left|Q_{23}Q_{24}\right|}-Z_{cr2}\sqrt{\left|Q_{23}Q_{24}\right|}-2Q_{25}}{2Q_{26}}, & z_{p3}=\;Z_{c2}+\;\sqrt{R_{2b}{}^{2}-{\left(x_{p3}-X_{c2}\right)}^{2}} \end{array}$$ (15)

$$\begin{array}{cc} x_{p4}=\;\frac{Q_{27}\sqrt{\left|Q_{28}Q_{29}\right|}-Z_{cr2}\sqrt{\left|Q_{28}Q_{29}\right|}-2Q_{30}}{2Q_{31}}, & z_{p4}=\;Z_{c3}+\sqrt{R_{3b}{}^{2}-{\left(x_{p4}-X_{c3}\right)}^{2}} \end{array}$$ (16)

The new back surface coordinates for the regions occupied by fillets are then defined as,

$${\text{Z}}_{\text{b}}=\{\begin{array}{cc} Z_{cr1}-\sqrt{r_{1}{}^{2}-{\left(x-X_{cr1}\right)}^{2}}, & x_{p1}\leq x<x_{p2}\\ Z_{cr2}+\sqrt{r_{2}{}^{2}-{\left(x-X_{cr2}\right)}^{2},} & x_{p3}\leq x\leq x_{p4} \end{array}$$ (17)

In all designs, the choice of base curve R_1b_, transient zone R_2b_ and peripheral zone radii R_3b_ was constrained such that,

$$R_{1b}=R_{2b}-0.5\;=R_{3b}-1\;\;(\text{all}\;\text{dimensions}\;\text{in}\;\text{mm})$$ (18)

The range of values used in the lens geometry design was chosen to cover the average dimensions of the commercially available contact lenses. Base curve radius (back optic zone radius), R_1b,_ was set to 8.20 mm, optic zone diameter d_1_ was set to 8.00 mm, and finally, the lens overall lens diameter, d_3_, was set to 14.50 mm and d_2_ to the mean value of d_1_ and d_3_.

**Table 2:** Lens back-surface shape parameters.

$Q_{1}=R_{1b}{}^{2}X_{c2}-R_{1b}{}^{2}X_{c1}+R_{2b}{}^{2}X_{c1}-R_{2b}{}^{2}X_{c2}-X_{c1}X_{c2}{}^{2}-X_{c1}{}^{2}X_{c2}+X_{c1}Z_{c1}{}^{2}+X_{c1}Z_{c2}{}^{2}+X_{c2}Z_{c2}{}^{2}+X_{c1}{}^{3}+X_{c2}{}^{3}+Z_{c1}$
$Q_{2}=R_{1b}{}^{2}+2R_{1b}R_{2b}-R_{2b}{}^{2}+X_{c1}{}^{2}-2X_{c1}X_{c2}+X_{c2}{}^{2}+Z_{c1}{}^{2}-2Z_{c1}Z_{c2}+Z_{c2}{}^{2}$
$Q_{3}=R_{1b}{}^{2}+2R_{1b}R_{2b}+4R_{1b}r_{1}+R_{2b}{}^{2}+4R_{2b}r_{1}-X_{c1}{}^{2}+2X_{c1}X_{c2}-X_{c2}{}^{2}-Z_{c1}{}^{2}+2Z_{c1}Z_{c2}-Z_{c2}{}^{2}+4r_{1}{}^{2}$
$Q_{4}=-R_{1b}{}^{2}+2R_{1b}R_{2b}-R_{2b}{}^{2}+X_{c1}{}^{2}-2X_{x1}X_{c2}+X_{c2}{}^{2}+Z_{c1}{}^{2}-2Z_{c1}Z_{c2}+Z_{c2}{}^{2}$
$Q_{5}=2X_{c1}Z_{c1}Z_{c2}-2X_{c2}Z_{c1}Z_{c2}-2R_{1b}X_{c1}r_{1}+2R_{1b}X_{c2}r_{1}+2R_{2b}X_{c1}r_{1}-2R_{2b}X_{c2}r_{1}$
$Q_{6}=X_{c1}{}^{2}-2X_{c1}X_{c2}+X_{c2}{}^{2}+Z_{c1}{}^{2}-2Z_{c1}Z_{c2}+Z_{c2}{}^{2}$
$Q_{7}=R_{1b}{}^{2}X_{cr1}-R_{1b}{}^{2}X_{c1}-X_{c1}X_{cr1}{}^{2}-X_{c1}{}^{2}X_{cr1}+X_{c1}Z_{c1}{}^{2}+X_{c1}Z_{cr1}{}^{2}+X_{cr1}Z_{c1}{}^{2}+X_{cr1}Z_{cr1}{}^{2}+X_{c1}r_{1}{}^{2}-X_{cr1}r_{1}{}^{2}+X_{c1}{}^{3}+X_{cr1}{}^{3}+Z_{c1}$
$Q_{8}=R_{1b}{}^{2}+2R_{1b}r_{1}-X_{c1}{}^{2}+2X_{c1}X_{cr1}-X_{cr1}{}^{2}-Z_{c1}{}^{2}+2Z_{c1}Z_{cr1}-Z_{cr1}{}^{2}+r_{1}{}^{2}$
$Q_{9}=-R_{1b}{}^{2}+2R_{1b}r_{1}+X_{c1}{}^{2}-2X_{c1}X_{cr1}+X_{cr1}{}^{2}+Z_{c1}{}^{2}-2Z_{c1}Z_{cr1}+Z_{cr1}{}^{2}-r_{1}{}^{2}$
$Q_{10}=X_{c1}Z_{c1}Z_{cr1}+X_{cr1}Z_{c1}Z_{cr1}$
$Q_{11}=X_{c1}{}^{2}-2X_{c1}X_{cr1}+X_{cr1}{}^{2}+Z_{c1}{}^{2}-2Z_{c1}Z_{cr1}+Z_{cr1}{}^{2}$
$Q_{12}=R_{2b}{}^{2}X_{cr1}-R_{2b}{}^{2}X_{c2}-X_{c2}X_{cr1}{}^{2}-X_{c2}{}^{2}X_{cr1}+X_{c2}Z_{c2}{}^{2}+X_{c2}Z_{cr1}{}^{2}+X_{cr1}Z_{c2}{}^{2}+X_{cr1}Z_{cr1}{}^{2}+X_{c2}r_{1}{}^{2}-X_{cr1}r_{1}{}^{2}+X_{c2}{}^{3}+X_{cr1}{}^{3}+Z_{c2}$
$Q_{13}=R_{2b}{}^{2}+2R_{2b}r_{1}-X_{c2}{}^{2}+2X_{c2}X_{cr1}-X_{cr1}{}^{2}-Z_{c2}{}^{2}+2Z_{c2}Z_{cr1}-Z_{cr1}{}^{2}+r_{1}{}^{2}$
$Q_{14}=-R_{2b}{}^{2}+2R_{2b}r_{1}+X_{c2}{}^{2}-2X_{c2}X_{cr1}+X_{cr1}{}^{2}+Z_{c2}{}^{2}-2Z_{c2}Z_{cr1}+Z_{cr1}{}^{2}-r_{1}{}^{2}$
$Q_{15}=X_{c2}Z_{c2}Z_{cr1}+\;X_{cr1}Z_{c2}Z_{cr1}$
$Q_{16}=X_{c2}{}^{2}-2X_{c2}X_{cr1}+X_{cr1}{}^{2}+Z_{c2}{}^{2}-2Z_{c2}Z_{cr1}+Z_{cr1}{}^{2}$
$Q_{17}=R_{2b}{}^{2}X_{c3}-R_{2b}{}^{2}X_{c2}+R_{3b}{}^{2}X_{c2}-R_{3b}{}^{2}X_{c3}-X_{c2}X_{c3}{}^{2}-X_{c2}{}^{2}X_{c3}+X_{c2}Z_{c2}{}^{2}+X_{c2}Z_{c3}{}^{2}+X_{c3}Z_{c2}{}^{2}+X_{c2}{}^{3}+X_{c3}{}^{3}-Z_{c2}$
$Q_{18}=-R_{2b}{}^{2}+2R_{2b}R_{3b}-R_{3b}{}^{2}+X_{c2}{}^{2}-2X_{c2}X_{c3}+X_{c3}{}^{2}+Z_{c2}{}^{2}-2Z_{c2}Z_{c3}+Z_{c3}{}^{2}$
$Q_{19}=R_{2b}{}^{2}+2R_{2b}R_{3b}-4R_{2b}r_{2}+R_{3b}{}^{2}-4R_{3b}r_{2}-X_{c2}{}^{2}+2X_{c2}X_{c3}-X_{c3}{}^{2}-Z_{c2}{}^{2}+2Z_{c2}Z_{c3}-Z_{c3}{}^{2}+4r_{2}{}^{2}$
$Q_{20}=X_{c2}Z_{c2}Z_{c3}+X_{c3}Z_{c2}Z_{c3}-R_{2b}X_{c2}r_{2}+R_{2b}X_{c3}r_{2}+R_{3b}X_{c2}r_{2}-R_{3b}X_{c3}r_{2}$
$Q_{21}=X_{c2}{}^{2}-2X_{c2}X_{c3}+X_{c3}{}^{2}+Z_{c2}{}^{2}-2Z_{c2}Z_{c3}+Z_{c3}{}^{2}$
$Q_{22}=R_{2b}{}^{2}X_{cr2}-R_{2b}{}^{2}X_{c2}-X_{c2}X_{cr2}{}^{2}-X_{c2}{}^{2}X_{cr2}+X_{c2}Z_{c2}{}^{2}+X_{c2}Z_{cr2}{}^{2}+X_{cr2}Z_{c2}{}^{2}+X_{cr2}Z_{cr2}{}^{2}+X_{c2}r_{2}{}^{2}-X_{cr2}r_{2}{}^{2}+X_{c2}{}^{3}+X_{cr2}{}^{3}+Z_{c2}$
$Q_{23}=R_{2b}{}^{2}+2R_{2b}r_{2}-X_{c2}{}^{2}+2X_{c2}X_{cr2}-X_{cr2}{}^{2}-Z_{c2}{}^{2}+2Z_{c2}Z_{cr2}-Z_{cr2}{}^{2}+r_{2}{}^{2}$
$Q_{24}=-R_{2b}{}^{2}+2R_{2b}r_{2}+X_{c2}{}^{2}-2X_{c2}X_{cr2}+X_{cr2}{}^{2}+Z_{c2}{}^{2}-2Z_{c2}Z_{cr2}+Z_{cr2}{}^{2}-r_{2}{}^{2}$
$Q_{25}=X_{c2}Z_{c2}Z_{cr2}+X_{cr2}Z_{c2}Z_{cr2}$
$Q_{26}=X_{c2}{}^{2}-2X_{c2}X_{cr2}+X_{cr2}{}^{2}+Z_{c2}{}^{2}-2Z_{c2}Z_{cr2}+Z_{cr2}{}^{2}$
$Q_{27}=R_{3b}{}^{2}X_{cr2}-R_{3b}{}^{2}X_{c3}-X_{c3}X_{cr2}{}^{2}-X_{c3}{}^{2}X_{cr2}+X_{c3}Z_{c3}{}^{2}+X_{c3}Z_{cr2}{}^{2}+X_{cr2}Z_{c3}{}^{2}+X_{cr2}Z_{cr2}{}^{2}+X_{c3}r_{2}{}^{2}-X_{cr2}r_{2}{}^{2}+X_{c3}{}^{3}+X_{cr2}{}^{3}+Z_{c3}$
$Q_{28}=R_{3b}{}^{2}+2R_{3b}r_{2}-X_{c3}{}^{2}+2X_{c3}X_{cr2}-X_{cr2}{}^{2}-Z_{c3}{}^{2}+2Z_{c3}Z_{cr2}-Z_{cr2}{}^{2}+r_{2}{}^{2}$
$Q_{29}=-R_{3b}{}^{2}+2R_{3b}r_{2}+X_{c3}{}^{2}-2X_{c3}X_{cr2}+X_{cr2}{}^{2}+Z_{c3}{}^{2}-2Z_{c3}Z_{cr2}+Z_{cr2}{}^{2}-r_{2}{}^{2}$
$Q_{30}=X_{c3}Z_{c3}Z_{cr2}+X_{cr2}Z_{c3}Z_{cr2}$
$Q_{31}=X_{c3}{}^{2}-2X_{c3}X_{cr2}+X_{cr2}{}^{2}+Z_{c3}{}^{2}-2Z_{c3}Z_{cr2}+Z_{cr2}{}^{2}$

Following the design of a two-dimensional lens profile, A three-dimensional back-surface profile was constructed in 1° steps, meridian by meridian. This final step was necessary for the use of lens geometry in both the finite element and light raytracing analysis.

### Peripheral-zone design

Unlike the back-surface, the front-surface was not rotationally symmetric. The asymmetric nature of the front surface meant that, when constructing the three-dimensional geometry, each meridian had to be considered individually. The thickness of the boundary between the transient zone and the periphery zone T_w_ was calculated in a way to allow the addition of thickness to certain meridians according to the type of balance zone.

$${\text{T}}_{{\text{w}}_{\text{i}}}=\{\begin{array}{cc} 0.4W_{i}(2T+Tc)/3 & \text{for}\;\text{G}1\text{P}\;\text{design}\\ 0.3W_{i}(2T+Tc)/3 & \text{for}\;\text{B}2\text{P}\;\text{design}\\ 0.2W_{i}(2T+Tc)/3 & \text{for}\;\text{B}4\text{P}\;\text{design} \end{array}$$ (19)

where T is the lens thickness at the end of the optical zone ($\text{X}=\frac{d_{1}}{2}$), *T*_*c*_ is the central thickness of the lens and *W* is a weighting factor defined as:

$$\text{W}=\{\begin{array}{cc} \text{cos}(\text{θ}+\frac{\text{π}}{2}) & \text{for}\;\text{G}1\text{P}\;\text{design}\\ \text{cos}(2\text{θ}) & \text{for}\;\text{B}2\text{P}\;\text{design}\\ cos(4\theta +\pi ) & \text{for}\;\text{B}4\text{P}\;\text{design} \end{array}$$ (20)

The weighting factor W was set to zero for meridian angles, θ, at which the calculated value was negative.

Finally, the lens edge thickness (T_e_) was set as a function of the optical power as 0.1+0.002|P_max_| before fitting the lens’s front-surface points via shape-preserving piecewise cubic interpolation [56] to ensure a smooth front-surface while keeping the designed points in their position. In this design configuration, P_max_ is either the summation of the spherical and cylindrical power or the spherical power only, whichever was higher.

The lens central thickness (T_c_) was then updated through an automatic loop to avoid producing regions of negative volume resulting from the intersection of the front- and back-surfaces during the lens design process. For each thickness change, R_1f_ and T_w_ were recalculated and the front-surface Z_f_ was updated accordingly.

## Appendix C: Material models

**Fig 12:**
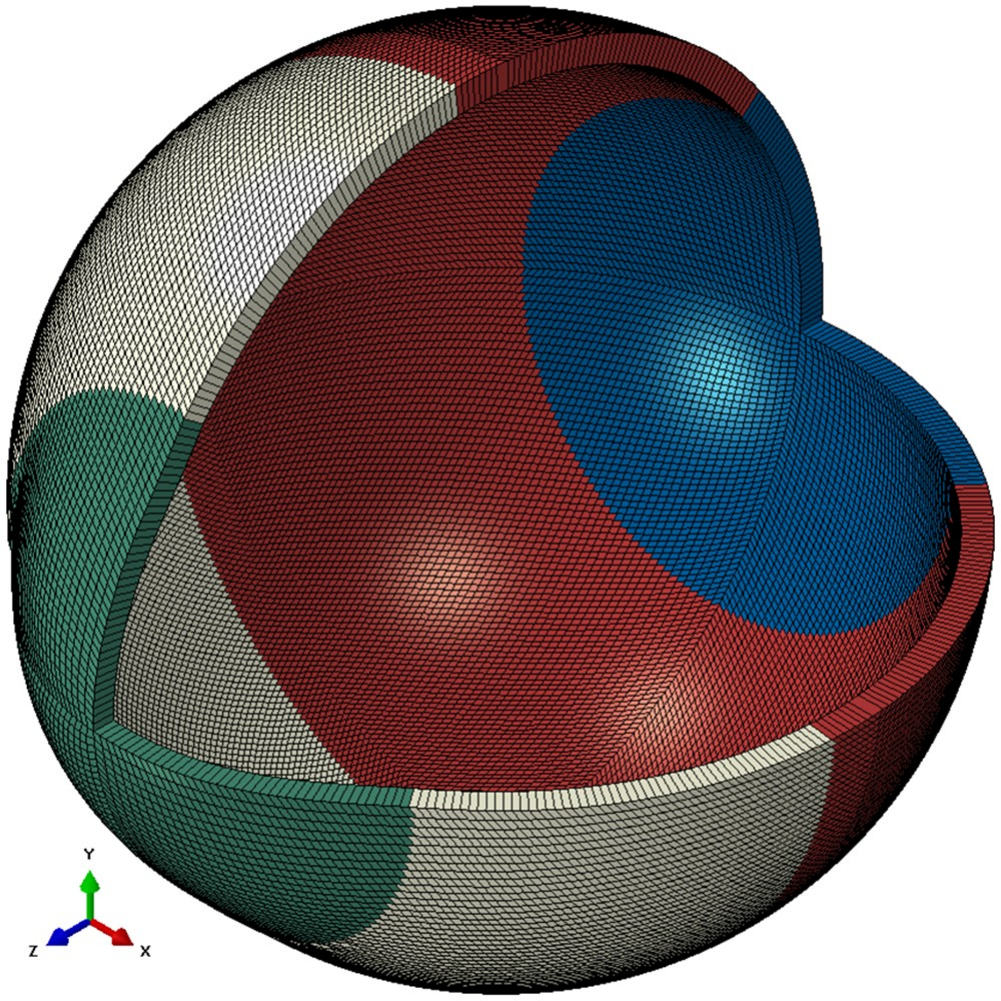
A typical FE model for the average eye used in this study where different colours represent different material models. The eye’s equatorial nodes were constrained in axial directions.

**Fig 13:**
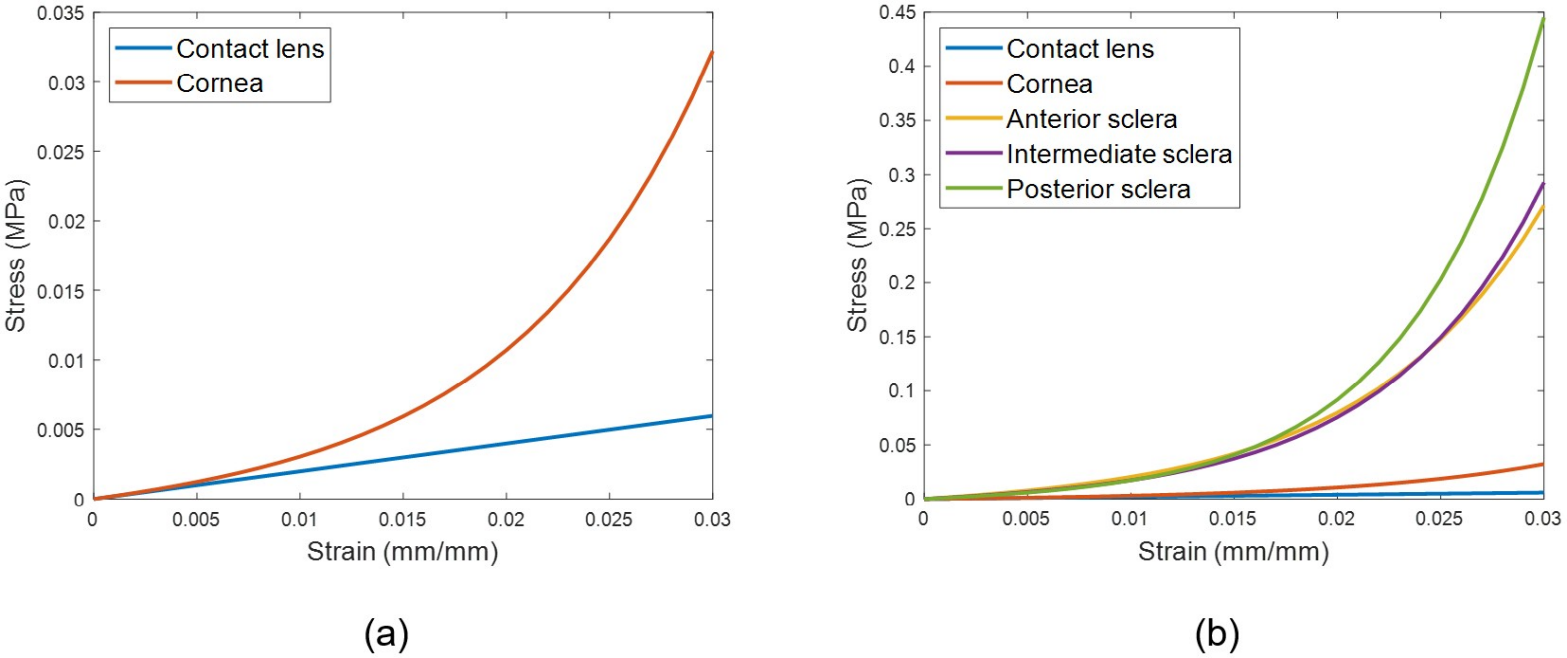
Stress-strain curves for the material models. Contact lens’s material was modelled as a linear elastic material, however, the eye was modelled as a hyper-elastic material.

The eye was modelled as hyper-elastic soft tissue with a water-like density of 1000 kg/m^3^ and four regions including the cornea (μ_c_ = 0.07, α_c_ = 110.8), anterior, intermediate and posterior sclera separated at elevation angles of 55°, 7.5°, -47.5° measured from the centre of the sclera [57], Fig 12. First-order Ogden material models [58] were used to represent the eye tissue’s mechanical performance with different stress-strain behaviour under loading conditions following earlier experimental studies [57, 59, 60], Fig 13. The purpose of splitting the sclera into three regions was to characterise regional mechanical properties of scleral tissue using circumferential regions of isotropic elements to replicate macroscale sclera displacements. Scleral materials were characterised as μ_s1_ = 0.441, α_s1_ = 124.5, μ_s2_ = 0.349, α_s2_ = 138.5, μ_s3_ = 0.308 and α_s3_ = 162.2.

Each contact lens from the three investigated soft contact lens designs (G1P, B2P & B4P) was modelled as an incompressible linear elastic solid with a Young’s modulus of 0.199 MPa, a Poisson’s ratio of 0.49 and the density of water 1000 kg/m^3^ [30].

## Supporting information

S1 File (ZIP)

S2 File (Z01)
